# Identification of rapidly induced genes in the response of peanut (*Arachis hypogaea*) to water deficit and abscisic acid

**DOI:** 10.1186/1472-6750-14-58

**Published:** 2014-06-26

**Authors:** Xiaoyun Li, Jiabao Lu, Shuai Liu, Xu Liu, Yingying Lin, Ling Li

**Affiliations:** 1 Guangdong Provincial Key Lab of Biotechnology for Plant Development, College of Life Science, South China Normal University, Guangzhou 510631, P R China; 2 Key Laboratory of Plant Resources Conservation and Sustainable Utilization, South China Botanical Garden, Chinese Academy of Science, Guangzhou 510650, P R China

**Keywords:** *Arachis hypogaea*, Transcriptome, Water deficit, Abscisic acid (ABA)

## Abstract

**Background:**

Peanut (*Arachis hypogaea*) is an important crop, but droughts often affect peanut production. There is a lack of genomic information available for peanut; therefore, little is known about the molecular basis of its drought stress response.

**Results:**

Previously, we found that peanut stomata close rapidly during water deficit and in response to abscisic acid (ABA) treatment, and many genes show changes in their expression levels. To screen for candidate genes involved in the water deficit response, we used the Illumina HiSeq2000/MiSeq sequencing platform to conduct a global transcriptome analysis of peanut seedlings under water deficit with or without an ABA pretreatment. Three peanut tissues (leaves, roots, and stems) collected at each of three developmental stages (four-leaf, flowering, and podding stages) were used to construct sequence libraries. Then, 4.96 × 10^7^ raw sequence reads were generated and the high quality reads were assembled into 47,842 unigenes. We analyzed these sequence libraries to identify differentially expressed genes (DEGs) under water deficit with or without ABA pretreatment. In total, 621 genes were induced rapidly (≥1.5 fold change compared with control) under water deficit, 2,665 genes were induced rapidly under water deficit + ABA pretreatment, and 279 genes overlapped between water deficit and water deficit + ABA pretreatment. Of the 279 overlapping genes, 264 showed the same expression pattern and 15 showed opposite expression patterns. Among the DEGs, 257 were highly induced (>5 fold) by water deficit + ABA pretreatment, while 19 were highly induced (>5 fold) by water deficit alone. The genes induced under water deficit + ABA pretreatment included 100 putative transcription factor (TF) genes, while those induced under water deficit alone included only 22 putative TF genes. To validate the transcriptome results, we conducted quantitative PCR (qPCR) analyses to quantify the transcript levels of nine candidate genes.

**Conclusions:**

The DEGs results show that many genes are rapidly induced in peanut in response to water deficit without or with ABA pretreatment. The results indicate that the main drought response mechanisms in peanut function through an ABA-dependent pathway. Our data provide a comprehensive sequence resource for molecular genetics research on peanut stress responses.

## Background

Different plant species have various physiological adaptations to deal with long-term or short-term drought stress. In the short-term drought stress response, the phytohormone abscisic acid (ABA) regulates guard cell movement to close stomata. However, ABA also functions in the long-term stress response by regulating the development of reproductive tissues via massive transcriptional reprogramming events under prolonged drought. This results in growth retardation and decreased crop yields [1,2]. In plants, drought resistance functions through ABA-dependent and ABA-independent regulatory systems, as revealed in studies on drought-induced genes in *Arabidopsis thaliana* and rice (*Oryza sativa*) [3]. In *Arabidopsis*, calcium-dependent and -independent pathways mediate transcriptional reprogramming via an ABA-dependent pathway under water deficit [3].

Peanut is one of the most important oil crops worldwide. It is widely cultivated in Asia, Africa, and the Americas [4]. However, seasonal droughts often affect peanut yields and quality, causing significant economic losses. Little is known about the molecular signaling and regulatory mechanisms of the drought response in peanut because of its complex genetic background, and the lack of systematic research. Therefore, we have focused our research on drought resistance of cultivated peanut for many years. In plants, water deficit triggers the expression of numerous stress-induced genes, which encode products that enhance drought tolerance and allow continued growth [5]. ABA plays a key role in this response, but ABA signaling components and target genes often overlap with other signaling systems [6]. Many drought-responsive transcription factor genes have been identified in various species, such as *AREB1/ABF2* and *DREB2A* in *Arabidopsis*, and *ABP9* in maize (*Zea mays*) [6-10]. In *Arabidopsis*, these drought-responsive transcription factors activate the expression of the downstream target genes *RD29A* and *RD29B*. Consequently, *RD29A* and *RD29B* can serve as marker genes for drought or ABA signaling [11,12]. Large-scale screening of peanut has identified some stress-related genes. For example, fungus-infected peanut seeds showed increased transcript levels of genes encoding late-embryogenesis-abundant (LEA) family proteins, and increased levels of their products [13]. Likewise, a drought-resistant relative of peanut, *Arachis duranensis*, showed increased transcript levels of basic leucine zipper (bZIP) transcription factor genes when subjected to drought (18% water content). A pest-resistant relative of peanut, *Arachis stenosperma,* showed increased transcript levels of MYB family transcription factor genes when infected with fungi [4].

In our previous studies, we cloned and characterized some drought-related genes from peanut, including the ABA synthesis gene *AhNCED1*[14,15], and three dehydration-induced transcription factor genes, *AhAREB1*[16], *AhNAC2*[17], and *AhNAC3*[18]. The expression of these genes rapidly responds to water deficit in peanut. Therefore, similar to *RD29A* and *RD29B* in *Arabidopsis*, the transcript level of *AhNCED1* can serve as a molecular marker for screening drought-resistant varieties of peanut. Furthermore, the promoter region of *AhNCED1* contains many abscisic acid-responsive elements (ABREs). Site-directed mutagenesis of two of these ABREs diminished *AhNCED1* promoter activity [19], suggesting that ABA signaling pathways directly regulate *AhNCED1*. The ABA-responsive element binding protein (AREB) gene *AhAREB1* was cloned from peanut and overexpressed in *Arabidopsis*[16]. A microarray analysis showed that the transcription of *AtNCED3* (the *Arabidopsis* homolog of *AhNCED1*) was significantly higher in the *AhAREB1*-overexpressing plants than in wild type [20]*.* Recently, we found that AhAREB1 may directly regulate *AhNCED1* (unpublished data).

The response to water deficit or ABA in *Arabidopsis* has been studied using transformation techniques, and the results of such studies have improved our understanding of the *Arabidopsis* drought response. However, similar research on the drought response of peanut has been hampered by the low efficiency of transformation and the complex tetraploid genome of this important crop plant. To address this issue, we used a global transcriptome analysis to study the mechanisms by which peanut copes with drought stress, and to provide a comprehensive sequence resource for further molecular genetic research on this species.

## Results and discussion

### Sequencing and *de novo* assembly

We generated 44,277,322 (4.41G) paired-end reads with an average length of 905 bp for the peanut cultivar Yueyou7. Of the clean reads, 95.96% had phred-like quality scores at the Q20 level and 88.33% had phred-like quality scores at the Q30 level, with GC contents of 48.09% and 49.45%, respectively. The high-quality reads were assembled into 47,842 unigenes (Table 1). We obtained 7,093,861 (0.35 G) clean reads from the water deficit without ABA-pretreatment group, 6,847,397 (0.34 G) clean reads from the water deficit + ABA-pretreatment group, and 7,486,557 (0.37 G) clean reads from the control (Table 1).

**Table 1:** Summary of short reads and assemblies

**Sample**	**Clean reads**	**Clean bases**	**GC (%)**	**Unigenes**	**Seq-dup-level (%)**
Peanut_R1	44277322	4.41G	48.09	47842	79.72
Peanut_R2	44277322	4.41G	49.45	47842	83.49
ABA_30min	6847397	0.34G	43.83	27969	45.2
PEG_30min	7093861	0.35G	48.43	26917	64.01
Control	7486557	0.37G	49.10	34837	72.6

Peanut_R1 represents left-reads, Peanut_R2 represents right-reads. Seq-dup-level (sequence duplication level) represents the proportion of repeated reads in total reads. ABA_30 min represents 30% PEG6000 treated for 30 min after ABA pretreatment for 30 min. PEG_30 min represents 30% PEG6000 treatment for 30 min. Control, no treatment.

### Genes modulated by water deficit and their predicted functions

**Figure 1:**
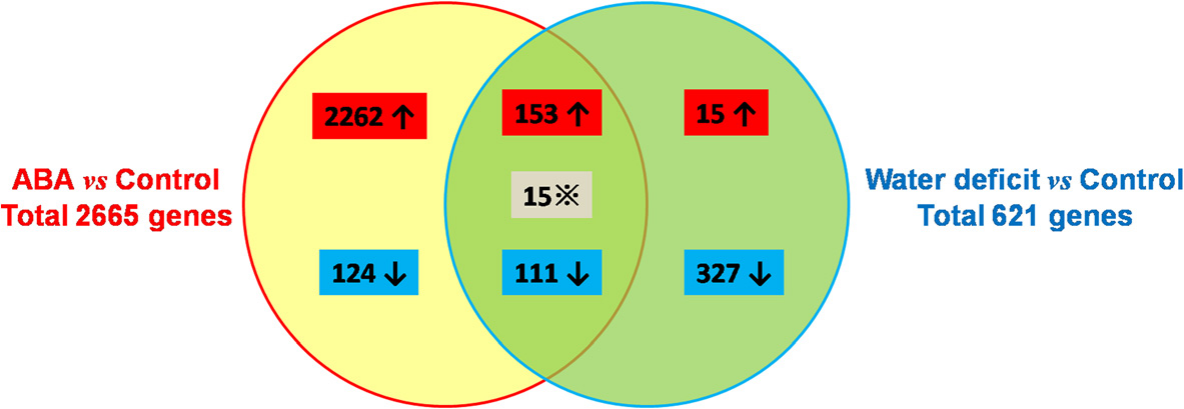
**Classification of ABA- and water deficit-inducible genes based on their expression patterns.** ABA- or water deficit-inducible up-regulated genes (red) or down-regulated genes (blue); intersection represents genes induced by both ABA and water deficit, “※” indicates genes showing opposite trends in expression between water deficit and ABA treatments.

To identify genes showing changes in expression in response to water deficit, peanut seedlings were subjected to a 30% PEG6000 treatment. This treatment has been used to simulate water deficit in several other studies [21,22]. The 30-min PEG treatment caused leaf wilting of 2-week-old peanut seedlings. After the PEG treatment, we collected leaves from the same position on the stalk for RNA extraction and sequencing. In total, 621 genes were rapidly induced (≥1.5 fold change compared with their respective expression levels in the control) by water deficit; 173 were up-regulated and 448 were down-regulated (Figure 1, Additional file 1). Also, 19 of these genes showed very large changes in expression levels (≥5-fold change compared with their respective expression levels in the control); 4 were up-regulated and 15 were down-regulated. Among the genes induced by water deficit alone, 22 encoded transcription factor-like proteins.

**Figure 2:**
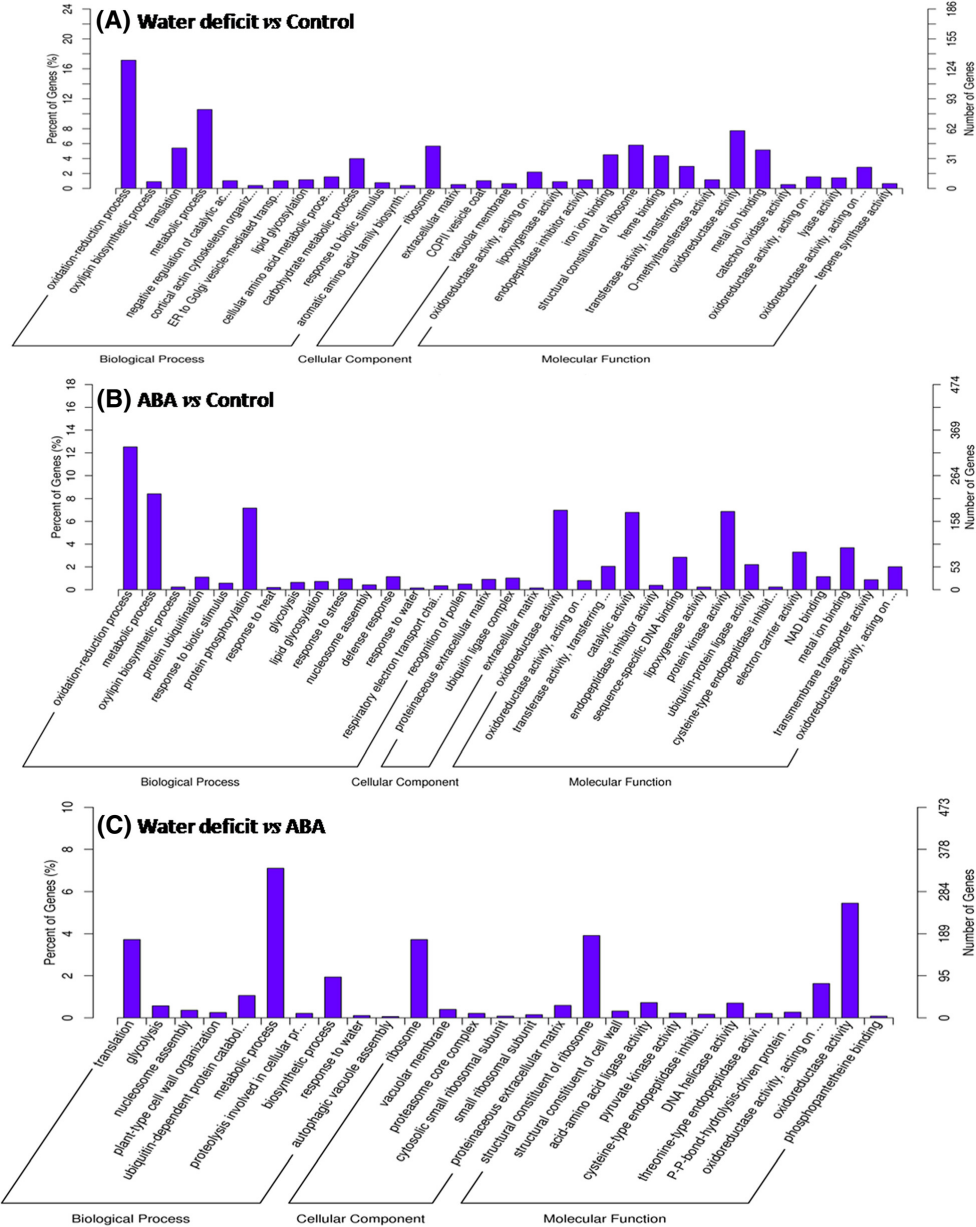
**GO annotation of DEGs in the transcriptome.** The DEGs were classified into different GO categories. **(A)** GO categories of differentially expressed genes response to water deficit, **(B)** GO categories of differentially expressed genes response to ABA, **(C)** GO categories of differentially expressed genes response to ABA or water deficit independently.

Next, we grouped the differentially expressed genes (DEGs) into GOseq functional categories (http://www.geneontology.org) based on Wallenius’s non-central hyper-geometric distribution [23]. The DEGs were grouped into three main categories (Figure 2); molecular function (42.91%), biological process (50.0%), and cellular components (2.19%). In a similar microarray study of the drought-resistant species *A. duranensis*, most contigs belonged to the molecular function category (49%), with fewer belonging to cellular component (26%) and biological process (39%) categories [4]. These findings suggest that the dynamic changes in gene expression differ between short-term and long-term drought responses.

We further analyzed each functional category to identify which proteins were the most enriched. Proteins related to oxidation reduction processes (17.14% genes) were the most enriched in the biological process category and oxidoreductases (7.73% genes) were the most enriched in the molecular function category. Ribosome (5.67% genes) was the most highly represented in the cellular components category. We used the KEGG (Kyoto Encyclopedia of Genes and Genomes) database to annotate the pathways and found that phenylpropanoid biosynthesis (90 genes) and linoleic acid metabolism (14 genes) were the most enriched pathways in peanut seedlings under water deficit (Table 2).

**Table 2:** KEGG annotations of enriched pathways in different transcriptomes

**Term**	**ID**	**Sample number**	**Background number**	**Q-Value**
**Water deficit *vs. *** **Control**				
Glycolysis/Gluconeogenesis	Ko00010	47	144	4.20 E-07
Plant-pathogen interaction	Ko04626	38	132	0.000218
**ABA *vs. *** **Control**				
Phenylpropanoid biosynthesis	Ko00940	21	90	2.7 E-06
Linoleic acid metabolism	Ko00591	14	45	8.01 E-06
**Water deficit *vs. *** **ABA**				
Glycolysis/Gluconeogenesis	Ko00010	62	144	0.003227
protein export	Ko03060	25	44	0.003227

### Genes modulated by water deficit + ABA pretreatment and their predicted functions

In peanut, ABA accumulation and distribution modulate physiological responses during the short-term response to water deficit [24,25]. In our preliminary experiments, exogenous ABA treatment caused stomata to close within 30 min, and ABA treatment for 30 min resulted in changes in the expression levels of numerous stress-related genes. In the present study, 2,665 genes showed altered expression (≥1.5 fold change compared with their respective expression levels in the control) in response to water deficit + ABA pretreatment; 2,425 genes were up-regulated and 240 genes were down-regulated (Figure 1, Additional file 1). Among these genes, those involved in gluconeogenesis (47 genes) and the plant–pathogen interaction (38 genes) were the most enriched (Table 2). Among the genes showing altered expression, 257 were highly induced (≥5 fold induction; 185 up-regulated, 75 down-regulated), and 100 genes encoded putative transcription factors (Additional file 2). The DEGs were classified into three main GO categories; molecular function (35.03%), biological process (2.08%), and cellular components (20.3%) (Figure 2). In the molecular function category, genes encoding proteins with oxido-reductase activity (6.98%), catalytic activity (6.79%) and protein kinase activity (6.87%) were the most enriched. Proteins related to oxidation-reduction processes (12.52%), metabolic process (8.42%), and protein phosphorylation (7.17%) were enriched in the biological process category. In the cellular components category, the proteinaceous extracellular matrix (0.91%) and ubiquitin ligase complex (1.02%) were the most highly enriched categories.

Many ubiquitin-related enzymes are thought to regulate the plant drought stress response, but for many of these candidate regulators, including *MMS21*[26] and *ZmRFP1*[27], their mechanism of action remains unclear. In *Arabidopsis,* AtPUB18 and AtPUB19 negatively regulate the ABA-mediated drought response [28,29]. In peanut, overexpression of the ubiquitin-related gene *AhUBC2* was shown to enhance drought tolerance [30]. Together, these results indicate that protein ubiquitination is linked to ABA-mediated drought responses in peanut. Compared with the water-deficit group, the water deficit + ABA pretreatment group showed dramatic down-regulation of water deficit-induced genes, including those related to translation, metabolic process, ribosome, structural constituents of ribosome, and oxido-reductase activity (Figure 2C).

### ABA-dependent and ABA-independent regulation of gene expression during the water deficit response in peanut

To examine the relationship between signaling under water deficit and that under water deficit + ABA pretreatment, we identified genes showing similar or different patterns of regulation between these two treatments. First, we identified 279 genes with significant overlaps in expression between the water deficit only and water deficit + ABA treatment groups. Of these, 153 genes were up-regulated and 111 were down-regulated by both water deficit alone and water deficit + ABA pretreatment, compared with their respective expression levels in the control. Only 81 genes were more strongly induced by water deficit + ABA treatment than by water deficit alone (>1.5 fold difference in water deficit + ABA group compared with their respective levels in water deficit only group). Of these 81 genes, 55 were up-regulated, 16 were down-regulated, and 12 encoded putative transcription factors.

The transcript levels of *comp65973_c0* (predicted to encode a MYC2-like transcription factor) decreased both under water deficit (–5.29 fold) and under water deficit + ABA pretreatment (–5.25 fold). The transcript levels of *comp64977_c0* (predicted to encode a NAC-like transcription factor) increased both under water deficit (2.45 fold) and under water deficit + ABA pretreatment (3.84 fold). The fact that water deficit + ABA pretreatment did not affect their expression more strongly than did water deficit alone suggests that ABA does not regulate the expression of these genes. Thus, these transcripts appear to be regulated by water deficit through an ABA-independent pathway.

Except for *comp64977_c0*, nine NAC or NAC-like genes showed rapid responses to water deficit + ABA pretreatment, but were not induced by water deficit alone. These genes, and the extent of their induction by water deficit + ABA, were as follows: *comp60831_c0/AhNAC3* (5.21 fold), *comp65819_c0/AhNAC2* (3.43 fold), *comp61624_c1* (1.81 fold), *comp65473_c0* (1.85 fold)*, comp66232_c1* (1.50 fold), *comp63320_c1* (3.55 fold), *comp65172_c0* (2.45 fold). These findings indicate that these genes are regulated by water deficit through an ABA-dependent pathway. *comp65819_c0/AhNAC2* and *comp60831_c0/AhNAC3* were reported to show significant increases in transcript levels under dehydration, water deficit (simulated by PEG), or ABA treatment [17,18]. We did not identify these loci as differentially expressed under water deficit in this experiment, probably because of the short treatment time (30 min) used in our analyses.

Among the overlapping genes, 15 showed differences in expression between water deficit and water deficit + ABA pretreatment; 10 were down-regulated under water deficit but up-regulated under ABA + water deficit, and 5 were up-regulated under water deficit but down-regulated under ABA + water deficit. This result indicates that these genes are regulated by water deficit through an ABA-dependent pathway, but they could have opposite functions in the water deficit response. For example, *comp66875_c0* and *comp62508_c0* were down-regulated by water deficit but up-regulated by ABA + water deficit. The protein encoded by *comp66875_c0* shows sequence similarity to a low-temperature-induced 65 kDa protein in *Glycine max*, and that encoded by *comp62508_c0* shows sequence similarity to the homeobox-leucine zipper protein ATHB-7. *comp62508_c0* was down-regulated by water deficit (–4.35 fold), but up-regulated by water deficit + ABA pretreatment (3.57 fold). In *Arabidopsis*, *ATHB-7* is transcriptionally regulated in an ABA-dependent manner and modulates ABA signaling by regulating the activities of PP2C and an ABA receptor gene [31,32]. Consistent with our results in peanut, in *Arabidopsis*, the *ATHB-7* response to ABA was observed in seedlings within 30 min of drought stress, and its transcript levels continued to increase after 12 h or 72 h of drought stress [31].

The genes encoding 9-cis-epoxycarotenoid dioxygenase (*comp69670_c1*/*AhNCED1*, 3.05 fold) and abscisic acid 8′-hydroxylase-like proteins (*comp54319_c0*, 4.44 fold; *comp65737_c0*, 1.64 fold), which regulate ABA biosynthesis and degradation, respectively, were significantly induced by water deficit + ABA pretreatment. In our previous study, we detected high transcript levels of *AhNCED1* in response to ABA or drought stress [14]. In the present study, all of the genes related to ABA metabolism showed increased transcript levels in response to exogenous ABA treatment, suggesting positive feedback regulation by ABA. However, the transcript levels of AREB-like transcription factors did not change in response ABA or water deficit, although many showed a constitutive high level of transcription under both conditions and in the control. Therefore, NAC transcription factors appear to respond quickly to water deficit + ABA pretreatment (ABA-dependent). These transcription factors, together with AREB-like transcription factors, may regulate ABA biosynthesis enzymes or hydroxylases. However, further experimental evidence is required to confirm this.

In previous studies, oligonucleotide array or cDNA microarray analyses have identified 200 ABA-responsive genes in rice [33] and 245 ABA-responsive genes in *Arabidopsis*[34]. More ABA-responsive genes were identified in peanut than in other plants. Here, 47,842 unigenes were obtained from the drought-resistant peanut cultivar Yueyou7. In another study, 7,723 high contigs were obtained from 2-month-old plants of *A. stenosperma* (V10309), and 12,792 high contigs were obtained from 3-month-old plants of *A. duranensis* (K7988) [4]. In a different study, 59,077 unigenes were obtained from 10 immature seeds of five plants [35]. These findings suggest that the distribution of unigenes differs among plant types to some extent.

We did not detect homologs of *RD29A* and *RD29B* in the peanut seedling transcriptome. We detected *comp66875_c0* and *comp56618_c0,* which encode proteins similar to the low-temperature-induced 65 kDa protein (accession numbers XP_003556105.1 and XP_003536469.1, respectively). Our results suggest that, similar to *RD29B*, *comp66875_c0* and *comp56618* are controlled mainly by ABA. *RD29A* and *RD29B* encode closely related hydrophilic proteins, and are quickly and strongly induced by drought and salt stress [5,36]. They are considered to be drought stress or ABA-signaling activity marker genes in *Arabidopsis*. Their promoters have been used to drive the expression of genes that confer water-stress tolerance, for example, wheat *expansin*[37] and rice *OsbZIP71*[38], without affecting plant growth and development. However, no close homologs of *RD29A* or *RD29B* have been identified in crop species such as rice, sorghum, maize, and soybean [12]. Therefore, *AhNCED1* and *comp66875_c0* represent marker genes for downstream events in the ABA-dependent pathway in peanut.

### Quantitative PCR validation of changes in gene expression

**Figure 3:**
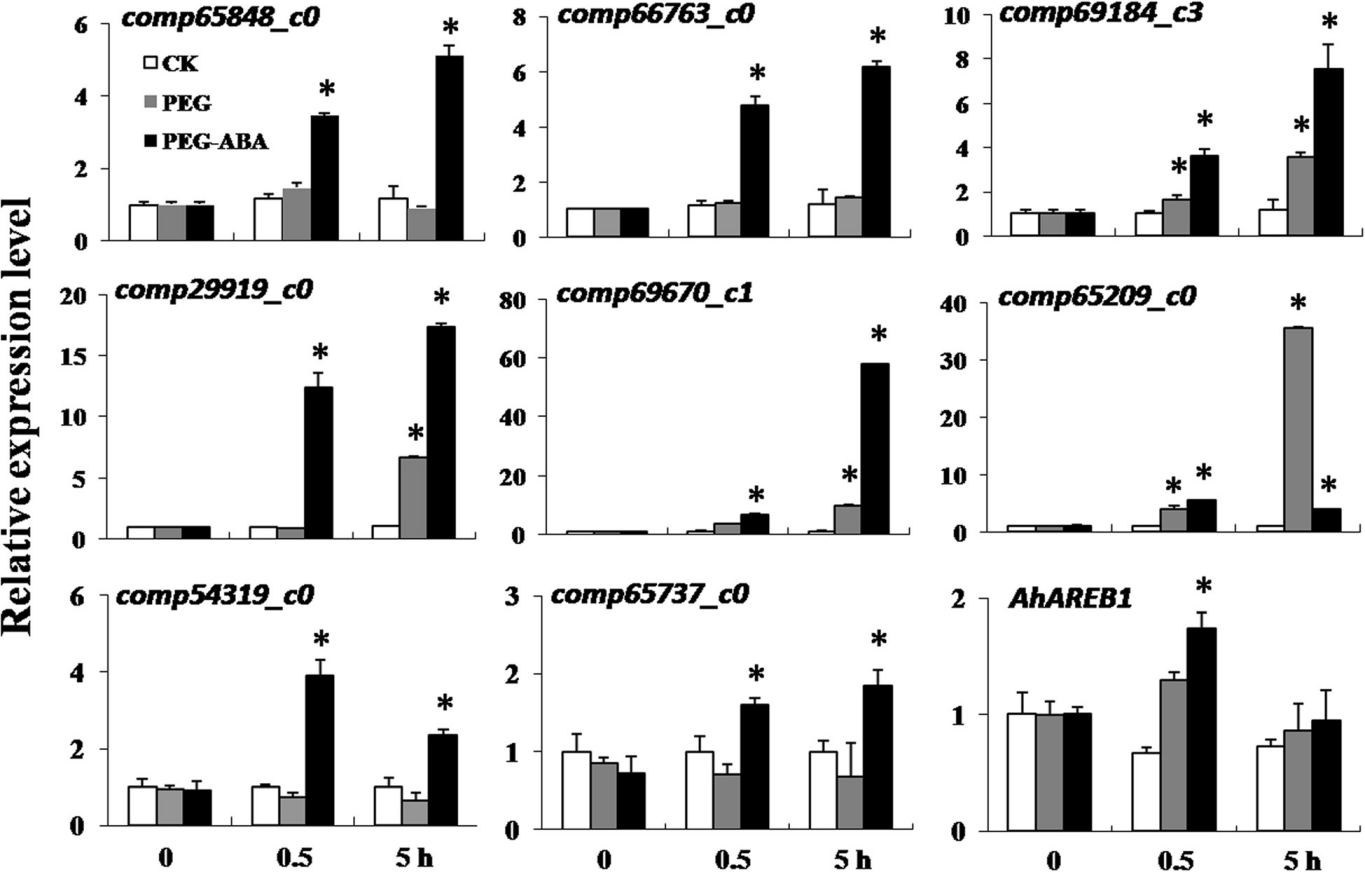
**Validation of transcript levels of nine candidate genes under water deficit and ABA by quantitative PCR (qPCR).***comp65848_c0* PREDICTED: histone deacetylase 5 like [*Glycine max*]; *comp66763_c0* PREDICTED: histone deacetylase 6 like [*G. max*]; *comp69184_c3* PREDICTED: WRKY transcription factor WRKY33 like [*G. max*]; *comp65209_c0* PREDICTED: MYB transcription factor MYB92 like [*G. max*]; *comp29919_c0* PREDICTED: putative transcription factor bHLH041 like [*G. max*]; *comp69670_c1* encodes nine-cis-epoxycarotenoid dioxygenase 1 [*Arachis hypogaea*]; *comp54319_c0* PREDICTED: abscisic acid 8-hydroxylase 3 [*G. max*]; *comp65737_c0* PREDICTED: abscisic acid 8-hydroxylase 4 [*G. max*]. * indicates significant difference (*P <* 0.05) between transcript level in PEG treatment group or PEG + ABA treatment group and that in CK (control).

To validate the gene expression changes detected in the transcriptome analysis, we used quantitative PCR to quantify the transcript levels of nine candidate genes. We analyzed the transcript levels in plants under water deficit and in those under water deficit + ABA pretreatment at 30 min and 5 h time points (Figure 3). *comp69184_c3*, *comp65209_c0,* and *comp29919_c0,* which encode putative transcription factors, were induced both by water deficit and water deficit + ABA pretreatment in our transcriptome analysis. Similar to the results of the transcriptome analysis, the qPCR results showed that all three of these genes were induced by water deficit or ABA at 30 min and 5 h. The quantitative PCR results also showed that *comp65209_c0* was strongly induced under water deficit at 5 h. *comp65848_c0*, *comp66763_c0*, *comp69670_c1*, *comp54319_c0*, and *comp65737_c0* were induced by ABA at 30 min or 5 h. These results were generally consistent with the results of our transcriptome analysis.

The transcript level of *comp69670_c1* increased under water deficit at 5 h, as reported previously. *comp69670_c1* (also known as *AhNCED1*) encodes a 9-cis-epoxycarotenoid dioxygenase; AhNCED1 is a key enzyme in ABA biosynthesis in peanut and its expression was shown to increase during drought stress [15,24,25]. *comp54319_c0* and *comp65737_c0,* which belong to the P450 family, are homologs of *abscisic acid 8-hydroxylase 3* and *abscisic acid 8-hydroxylase 4* from soybean (*Glycine max*), respectively, suggesting that they encode enzymes involved in ABA catabolism. ABA is rapidly synthesized and accumulates under water deficit to induce stress resistance in plants. However, stress responses also include a protection mechanism to avoid excessive ABA accumulation, and this can retard growth. Thus, ABA catabolism genes are rapidly induced after ABA accumulation. The biosynthesis and catabolism genes play major roles in maintaining a balance of endogenous ABA for stress resistance and growth recovery after drought stress.

In our previous study, we found that *AhAREB1* was induced by ABA or water deficit [16,20]. We did not detect significant expression of *AhAREB1* under water deficit or ABA in our transcriptome data, so we revalidated its expression. We found that *AhAREB1* expression significantly increased after 0.5 h water deficit with ABA pretreatment, but not under water deficit, although it was slightly up-regulated. The different expression patterns detected in our studies may be due to experimental errors in treating plant materials or in transcriptome sequencing. This highlights the importance of validating the results of large-scale transcriptome analyses.

## Conclusion

We used RNA-Seq to conduct a global characterization of the peanut transcriptome response to water deficit and ABA. We generated 4.96 × 10^7^ raw reads, assembled into 47,842 unigenes, from the peanut cultivar Yueyou7*.* We identified DEGs between the early response to water deficit or water deficit + ABA pretreatment. These genes were annotated with GO functional categories; under water deficit there were 33 categories, and under water deficit + ABA pretreatment there were 31 categories. Only 19 genes were highly induced by water deficit, but 257 genes were highly induced by water deficit + ABA pretreatment. Now that these genes have been identified, our future research will focus on their functions and relationships. These data will be useful for functional genomic studies on peanut. We have established a biotechnological platform to study the early drought- and ABA-induced transcriptome regulatory network in peanut.

## Methods

### Plant growth and treatments

We used the South China peanut *(Arachis hypogaea* L.) cultivar Yueyou7 in this study. Yueyou7 is a commercial tetraploid line provided by the Crops Research Institute, Guangdong Academy of Agricultural Sciences (GDAAS), China. This cultivar is resistant to drought, rust, and bacterial infection. At present, there is no reference genome available for peanut, because its entire genome has not yet been sequenced. Therefore, we constructed a sequence library for Yueyou7. Then, we analyzed the sequence library to determine the profiles of DEGs under three different treatments; control, water deficit without ABA, and water deficit + ABA. In our previous study, we found that stomata closed after a 30-min 30% PEG treatment, indicative of a rapid stress response (regulation of stomatal closure and plant adaptation to water deficit) in the leaves [1]. Therefore, in this study, the profiles of DEGs in peanut leaves were determined after subjecting seedlings to water deficit or ABA treatments for 30 min.

To construct the sequencing library, plants were grown under normal conditions as described previously [14,17]. Seeds were planted in soil, and grown in a greenhouse at 25–30°C with ample irrigation. Three tissues (leaves, roots, and stems) were collected at three development stages (four-leaf, flowering, and podding stages). Then, all of these tissues were mixed to extract total RNA for sequence library construction. For the DEGs study, 2-week-old plants were divided into three groups, each with three independent replicates. The three groups were as follows: (1) water deficit without ABA (seedlings immersed in 30% PEG6000 solution for 30 min); (2) water deficit + ABA (seedlings immersed in 100 μmol/L ABA for 30 min, then in 30% PEG6000 solution for 30 min); (3) control (no treatment). All treatments were conducted in parallel. After treatments, total RNA was extracted from 100 mg leaf tissue, and RNA integrity was checked by gel electrophoresis. The quality and quantity of RNA were evaluated using an Agilent 2100 Bioanalyzer (Agilent technologies, Santa Clara, CA, USA) and a Nanodrop ND-1000 (Thermo Scientific, Waltham, MA, USA).

### *De novo* assembly and analysis of Illumina reads

The raw data (8 G) were pre-processed as previously described [4,38]. First, the adapter sequences, poor-quality sequences containing N (unknown sequences), and low-quality reads were filtered out to obtain clean reads. The clean reads were then combined to form longer fragments. All unigenes were compared with the NR (NCBI non-redundant protein sequences), NT (NCBI non-redundant nucleotide sequences), Pfam (protein family), and KO (KEGG Ortholog) databases using BLASTX v2.2.14. Based on the results of the protein database annotation, blast2GO was used to obtain the functional classifications of the unigenes based on GO terms. All transcription data have been uploaded into the Gene Expression Omnibus (http://www.ncbi.nlm.nih.gov/geo/query/acc.cgi?acc=GSE56439).

### Analysis of differences in gene expression

Because there is no high-quality genome sequence available for peanut, we used RSEM (RNA-Seq by Expectation Maximization) software to map and quantify the transcriptome based on our extensive sequence library as described above. We mapped and quantified 87.11%, 91.14%, and 93.09% of reads in the control, ABA, and PEG treatment groups, respectively. Then, we used RPKM (reads per kilobase per million mapped reads) measures to estimate gene expression levels [39]. Roughly, the genes belonged to high- (RPKM > 15), medium- (3.57 < RPKM < 15) or low-level (0.1 < RPKM < 3.57) expression groups. Finally, DEGseq (an R package) was used to identify DEGs using the RNA-seq data from different samples, as described previously [40].

### Validation of transcriptome data using quantitative PCR

To validate the results and provide a basis for further transcriptome analysis, nine genes were selected and their transcript levels were analyzed by qPCR. Total RNA was isolated by TRIzol reagent (Invitrogen, Carlsbad, CA, USA). Two-step real-time RT-PCR was performed with an SYBR Premix Ex Taq II kit (Takara, Dalian, China), and qPCR was performed using an Optical 96-well Fast Thermal Cycling Plate with an ABI 7500 real-time PCR system. Each reaction consisted of 10 μL 2 × SYBR Premix Ex Taq, 20 ng cDNA, and 0.1 μmol/L gene-specific primers in a final volume of 20 μL. The thermal cycling conditions were as follows: 95°C for 30 s, then 40 cycles at 95°C for 5 s, and 60°C for 34 s. Gene-specific primers are listed in Additional file 3. The *18S rRNA* gene was used as the internal control as previously described [16,20]. The relative expression levels were calculated using the relative 2^-ΔCt^ method as described previously [20].

### Availability of supporting data

The data sets supporting the results of this article are included within the article and its additional files.

The sequence data sets supporting the results of this article are available in the Gene Expression Omnibus repository [http://www.ncbi.nlm.nih.gov/geo/query/acc.cgi?acc=GSE56439].

## Competing interests

The authors declare that they have no competing interests.

## Authors’ contributions

LXY, LX and LL devised the method and designed the study. LXY, LJB, LS and LYY carried on the experimental work. LXY and LJB analyzed the data. LXY and LX wrote the manuscript. All authors contributed to the manuscript. All authors read and approved the final manuscript.

## Supplementary Material

Additional file 1 **Differentially expressed genes.** List of genes identified as ABA vs CK (control), water deficit vs CK, water deficit VS ABA, and similar or opposite trend, respectively, with a fold-change of at least 1.5. All differential genes were compared with the NR (NCBI non-redundant protein sequences), NT (NCBI non-redundant nucleotide sequences), Pfam (protein family), and KO (KEGG Ortholog) databases. Click here for file

Additional file 2 **Putative transcription factor genes expressed in peanut in response to ABA and water deficit.** List of genes differentially regulated by ABA and water deficit in peanut that were identified as putative transcription factor genes. Click here for file

Additional file 3 **Primers used in this study.** List of primers and oligonucleotides used for qRT-PCR. Click here for file
